# Author Correction: A comprehensive update to the *Mycobacterium tuberculosis* H37Rv reference genome

**DOI:** 10.1038/s41467-022-35402-2

**Published:** 2022-12-07

**Authors:** Poonam Chitale, Alexander D. Lemenze, Emily C. Fogarty, Avi Shah, Courtney Grady, Aubrey R. Odom-Mabey, W. Evan Johnson, Jason H. Yang, A. Murat Eren, Roland Brosch, Pradeep Kumar, David Alland

**Affiliations:** 1 Ray V. Lourenco Center for the Study of Emerging and Re-emerging Pathogens, Rutgers University—New Jersey Medical School, Newark, NJ USA; 2 Public Health Research Institute, Rutgers University—New Jersey Medical School, Newark, NJ USA; 3 Department of Pathology, Immunology and Laboratory Medicine, New Jersey Medical School, Rutgers—The State University of New Jersey, Newark, NJ USA; 4 Department of Medicine, University of Chicago, Chicago, IL USA; 5 Committee on Microbiology, University of Chicago, Chicago, IL USA; 6 Department of Microbiology, Biochemistry and Molecular Genetics, Rutgers University—New Jersey Medical School, Newark, NJ USA; 7 Division of Computational Biomedicine, Boston University School of Medicine and Bioinformatics Program, Boston University, Boston, MA USA; 8 Bioinformatics Program, Boston University, Boston, MA USA; 9 Center for Data Science, Rutgers University—New Jersey Medical School, Newark, NJ USA; 10 Helmholtz Institute for Functional Marine Biodiversity (HIFMB), Oldenburg, Germany; 11 Bay Paul Center, Marine Biological Laboratory, Woods Hole, MA USA; 12 Institut Pasteur, Université Paris Cité, Unit for Integrated Mycobacterial Pathogenomics, CNRS UMR 6047 Paris, France

**Keywords:** Bacterial genetics, Bacterial genomics, Pathogens, Genome assembly algorithms

Correction to: *Nature Communications* 10.1038/s41467-022-34853-x, published online 18 November 2022

The original version of the Supplementary Information associated with this Article was missing a list of Supplementary References. The HTML has been updated to include a corrected version of the Supplementary Information.

## Supplementary information

Updated Supplementary Information

