# Supplementary material for: A comprehensive update to the Mycobacterium tuberculosis H37Rv reference genome
Source: Nat Commun. 2022 Nov 18;13:7068. doi: 10.1038/s41467-022-34853-x (PMC9673877; doi:10.1038/s41467-022-34853-x)
Supplement: Supplementary file 1 — Supplementary information [file 41467_2022_34853_MOESM1_ESM.pdf]

A comprehensive update to the *Mycobacterium tuberculosis* H37Rv reference genome

Supplementary Information

Poonam Chitale, Alexander D. Lemenze, Emily C. Fogarty, Avi Shah, Courtney Grady, Aubrey R. Odom-Mabey, W. Evan Johnson, Jason H. Yang, A. Murat Eren, Roland Brosch, Pradeep Kumar, David Alland

SUPPLEMENTARY NOTES

Supplementary Note 1. Developing Bact-Builder with *in silico* reads

As part of Bact-Builder development and performance testing of individual steps, we generated simulated long and short reads off the H37Rv (NC_000962.3) linear reference genome. data using BadReads[1] and ART [2], respectively. These reads provided us with consistent high quality input data for initial validation and comparative analysis of robustness and reproducibility among various optional tools in the pipeline, allowing us to remove common experimental variables that can affect sequencing output and quality (ex. gDNA and sequencing library quality). We compared four commonly used long read assembly tools: Canu, Flye, Miniasm, and Raven, used in triplicate to assemble *in silico*-generated long read sequences. We found that each assembler produced a single linear contig, although the contig size varied across different assemblers (Figure S1 a-b). This contig was not circularized as the *in silico* reads were generated off a linear genome. Next, in order to determine the degree of sequencing coverage required for optimal assemblies, our *in silico* data was subsetted using Rasusa [3] into individual read sets containing 30x, 50x, 100x, 250x, and 500x coverage. Previous studies have demonstrated that bacterial genomes can be fully assembled using as little as 30x long read sequencing coverage [4–6], however bacteria with high GC content often require higher coverage [7]. Testing our assembler against *in silico* generated reads, we determined that coverage higher than 30x did not lead to significant improvements in long read assemblies (Figure S1b).

Further comparative analysis of assembler differences using anvio [8] revealed that these size differences did not substantially change annotations across any of the individual assemblies, apart from Miniasm which was missing 2 gene clusters observed in other assemblies. However, a further comparison between individual assemblers using DNAdiff [9] revealed differences in SNP and indel counts and several regions of difference when compared to the H37Rv1998 reference, even though the reads were created using the same reference.

This *in silico* data analysis revealed inherent assembly differences associated with specific assemblers, suggesting the need for a better standard assembly tool that would consistently and correctly reconstruct microbial genomes. We attempted to reconcile the differences observed across assemblers using Trycycler [10] to generate a consensus sequence from the outputs of the four individual assemblers, and further improve it by polishing steps using both long- and short- reads. Using *in silico* generated long reads, Trycycler generated a consensus assembly that was 4,411,524 bases which was closer in size to the H37Rv reference (4,411,532 bp) than most individual assemblies (Table S3, Figure S1 a-b). We next evaluated the optimal approach for final polishing steps, examining several iterations of both long and short read polishing tools (Table S2). An evaluation of polishing output, ease of use and amount of upstream data manipulation was used to determine the final workflow of three rounds of Racon (https://github.com/isovic/racon) using long read data, followed by one round of Medaka (https://github.com/nanoporetech/medaka) with long read data and finally three rounds of Pilon[11] polishing using Illumina data. Nextflow logs detail statistics on CPU usage, memory, job duration and input/output for *in silico* analysis (Figure S2). The *in silico* data based assembly did not require base calling or demultiplexing, and the remaining steps: assembly, Trycycler and polishing took approximately 15.8 hours on a standard CPU node to complete (Figure S2 a-d).

Supplementary Note 2. Evaluating individual polished assemblies against Bact-Builder

In order to further evaluate individual assemblers relative to the Bact-Builder output, each assembly that went into Trycycler was polished using the polishing steps of Bact-Builder (Racon (x3), Medaka (1x), and then Pilon (x3)). The resulting genomes indicated that polishing significantly improves individual assembler output but that there were still size and indel differences between the polished assemblies (Table S4). Canu assemblies in particular were larger in size than any other assembler and were on average 25,000bp larger than H37Rv.1 (Table

S4). Closer examination of the Canu differences showed large duplications at the start/end of the genome. These results highlight another benefit of using Bact-Builder.

We further observed that 7 out of the 12 polished assemblies contained the same one bp C insertion compared to our H37Rv.1 sequence (Table S5). ONT and illumina data was mapped to H37Rv.1, Canu 1 and Canu 2 polished assemblies using minimap2 and bowtie2 respectively. Canu 1 and Canu 2 were chosen for read alignment because the indels found in those assemblies represented all of the indels found across all 12 assemblies (Table S5). Evaluation of ONT read coverage at Canu 1 1,118,735insC and Canu 2 3,583,601insC, which were the same basepair deleted in H37Rv.1, indicated that the insertion was truly present in the reads and likely not the result of a minor variant present in the sample (Figure S5). Furthermore, evaluation of the illumina paired end reads indicated that there was sufficient coverage at the indel site (Figure S5) indicating that the base was truly present in the majority of the sample and again not the result of a minor variant.

We also evaluated the other identified indels found in the Canu 2 assembly relative to H37Rv.1 (Table S5). Two tandem base insertions: 2,313,498insG and 2,313,499insA were present were present in multiple reads in the Canu 2 ONT assemblies; however, only 7% of the illumina reads aligned to this region showed these insertions (Figure S5). Given the almost complete lack of illumina coverage for these Canu 2 insertions, as well as the absence of these insertions in the Canu 1 and Canu 3 assemblies, the evidence indicates that these two insertions are assembly errors rather than true minor variants. Furthermore, analysis of ONT and illumina read coverage at H37Rv 104,106insG, revealed that the base was truly present in the sequence and that the deletion in Canu 2 was an assembly error and not a low frequency variant (Figure S5).

In order to address the incorrectly called indel in H37Rv.1, the Guppy basecaller and relevant assemblers were updated to the latest software version at the time of writing.

Supplementary Note 3. Evaluating updated programs used within Bact-Builder

In order to evaluate the impact of relevant software updates on Bact-Builder, we updated the Guppy basecaller (v4.2.2 -> 6.0.1), Canu (v1.5 ->2.0) and Flye (v2.8 -> 2.9) assemblers used in Bact-Builder v1.0 with the latest component releases (at the time of writing (8/22/22)) and compared the outputs to what we had observed with previous versions of this software used in all of our prior analyses. (Figure S6). We saw that individual assemblers still produced genomes of varying sizes and that polishing was still required to generate an accurate assembly. Importantly, the updated Bact-Builder pipeline (BactBuilder v1.1) produced a H37Rv genome that was identical to H37Rv.1 in structure produced by the original Bact-Builder (Bact-Builder v1.0) and differed by a single insertion, correcting the previously identified deletion in H37Rv.1. The updated sequence and annotated file is available in the supplementary materials (Supplementary Data 2 and 3), and has been deposited to Genbank (accession # CP110619).

Table S1. Library prep protocols for Illumina and ONT sequencing			
**Company**	**Kit Name**	Minimum Input required	Source
Illumina	PCR-free Tagmentation	25-300 ng	https://www.Illumina.com/products/by-type/sequencingkits/library-prep-kits/truseq-dna-pcr-free.html
ONT (MinION)	Ligation Sequencing Kit	1000ng	https://store.nanoporetech.com/us/sample-prep/ligationsequencing-kit.html

Table S2. Various polishing tools and combinations evaluated for Bact-Builder pipeline				
Name	# of contigs	Size (bp)	SNP count relative to H37Rv reference	Indel count relative to H37Rv reference
Racon (illumina)x3 + Medaka (ONT) + Pilon (illumina)x3	1	4411530	0	2
Racon (illumina)x3 + Medaka (ONT)	1	4411402	0	134
Racon (ONT)x3 + Medaka (ONT) + Pilon (illumina)x3	1	4411530	0	2
Medaka (ONT) + Pilon (illumina)x3	1	4411530	0	2
*SNP: Single Nucleotide Polymorphism; ** Indels: single base insertions or deletions				

Table S3. *In silico* assembly results and DNAdiff analysis results comparing individual assemblies to the H37Rv reference (H37Rv ref)					
Name	# of contigs	Size (bp)	SNP* count (relative to ref)	Indel** count (relative to ref)	Regions of difference (relative to ref)
H37Rv ref	1	4411532	-	-	-
H37Rv *in silico* canu1	1	4411506	0	0	2
H37Rv *in silico* canu2	1	4411507	0	0	2
H37Rv *in silico* canu3	1	4411270	0	0	2
H37Rv *in silico* flye1	1	4411531	0	0	1
H37Rv *in silico* flye2	1	4411531	0	0	1
H37Rv *in silico* flye3	1	4411531	0	0	1
H37Rv *in silico* miniasm 1	1	4411567	4	118	1
H37Rv *in silico* miniasm 2	1	4411533	2	101	2
H37Rv *in silico* miniasm 3	1	4411564	10	114	2
H37Rv *in silico* raven1	1	4411460	1	4	2
H37Rv *in silico* raven2	1	4411394	0	5	2
H37Rv *in silico* raven3	1	4411465	0	4	2
H37Rv *in silico* Trycycler	1	4411524	0	0	2
H37Rv *in silico* Trycycler + Polish	1	4411524	0	0	2
*SNP: Single Nucleotide Polymorphism; ** Indels: single base insertions or deletions					

Table S4. SNP and indel count of polished individual assemblies							
Assembly	Size (bp)	SNP* compared to H37Rv ref	SNP* compared to H37Rv.1	Indel** compared to H37Rv ref	Indel** compared to H37Rv.1	Region of difference relative to H37Rv.1	Region of difference relative to H37Rv ref
H37Rv1998	4411532	n/a	109	n/a	36	10	0
H37Rv.1	4417941	109	0	36	0	0	10
Canu 1	4440440	109	0	35	0	1	11
Canu 2	4444608	109	0	38	4	1	11
Canu 3	4443581	109	0	36	0	1	11
Flye 1	4417941	109	0	36	0	1	11
Flye 2	4417942	109	0	35	1	1	11
Flye 3	4417934	109	0	35	1	1	11
Miniasm 1	4417942	109	0	35	1	1	11
Miniasm 2	4417941	109	0	36	0	1	11
Miniasm 3	4417942	109	0	37	1	1	11
Raven 1	4417934	109	0	35	1	1	11
Raven 2	4417942	109	0	35	1	1	11
Raven 3	4417934	109	0	36	0	1	11
*SNP: Single Nucleotide Polymorphism; ** Indels: single base insertions or deletions							

Table S5. Indel position in individual polished assemblies relative to H37Rv.1				
Assembly	Position in H37Rv.1	Basepair in H37Rv.1	Basepair in polished assembly	Position in Polished assembly
Canu 1	3251952	.	C	1118735
Canu 2	104106	G	.	2313506
	104113	.	G	2313498
	104113	.	A	2313499
	3251952	.	C	3583601

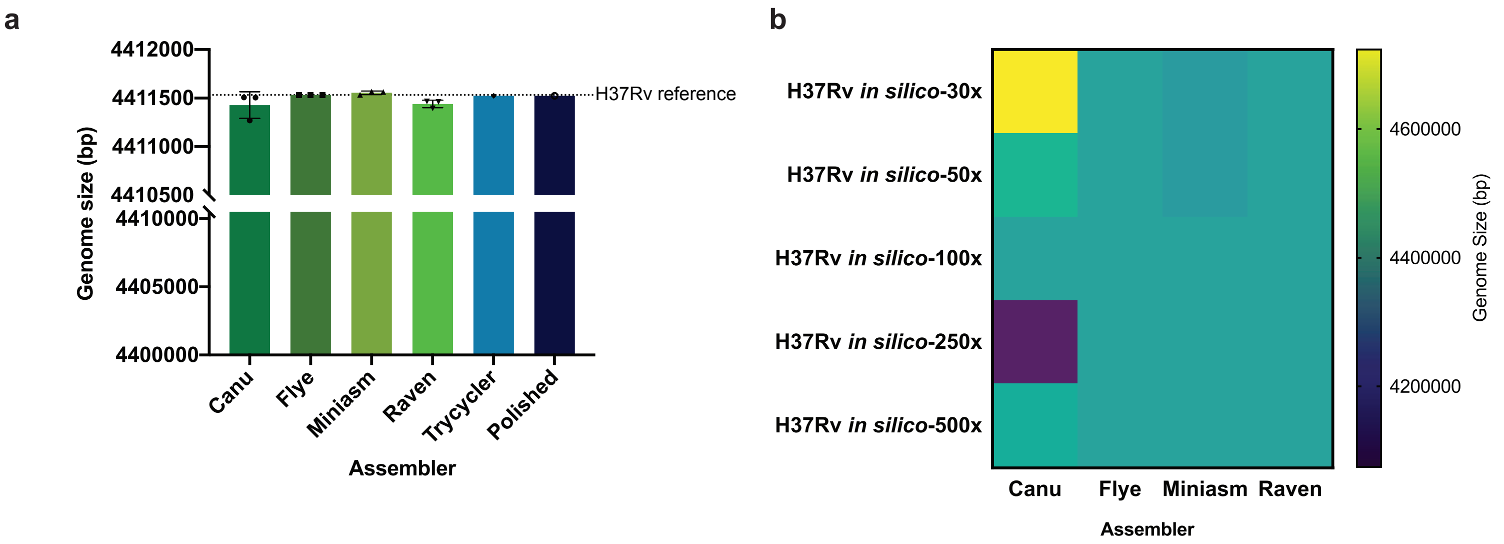

Figure S1. Developing Bact-Builder a. Comparison of assembly sizes generated by each assembler using *in silico* created reads. All assemblers were run in triplicate as stated in the Bact-Builder methods and resulting data is plotted as mean + SD. **b**. Heatmap comparison of genome sizes of the four *de novo* long read assemblers of using *in silico* created sequencing reads. The sequence coverage sampled for each analysis is shown in each row on the Y axis.

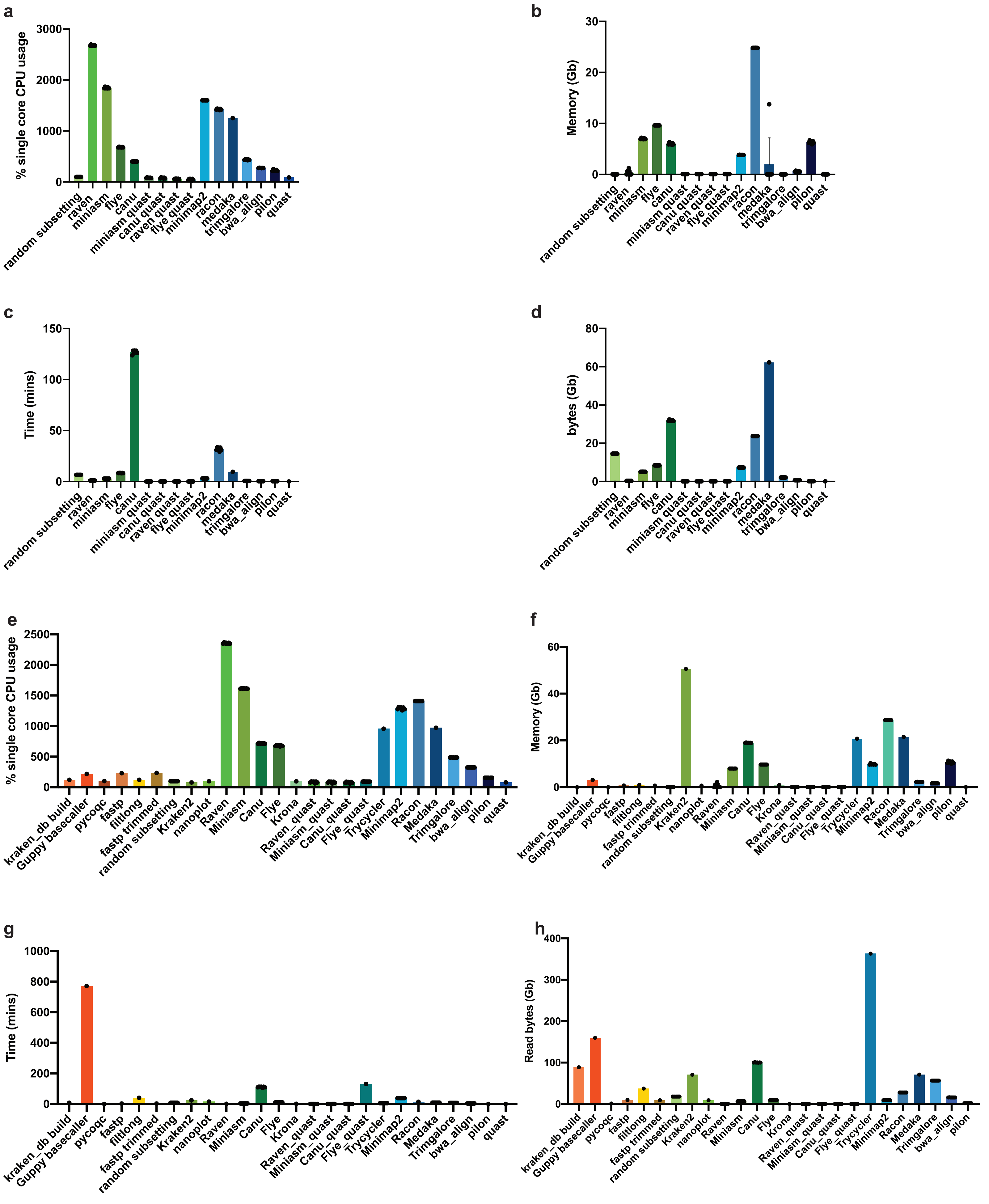

a
% single core CPU usage
0
1000
2000
3000
random subsetting
raven
miniasm
flye
canu
miniasm quast
canu quast
raven quast
flye quast
minimap2
racon
medaka
trimgalore
bwa_align
pilon
quast
b
Memory (Gb)
0
10
20
30
random subsetting
raven
miniasm
flye
canu
miniasm quast
canu quast
raven quast
flye quast
minimap2
racon
medaka
trimgalore
bwa_align
pilon
quast
c
Time (mins)
0
50
100
150
random subsetting
raven
miniasm
flye
canu
miniasm quast
canu quast
raven quast
flye quast
minimap2
racon
medaka
trimgalore
bwa_align
pilon
quast
d
bytes (Gb)
0
20
40
60
80
random subsetting
raven
miniasm
flye
canu
miniasm quast
canu quast
raven quast
flye quast
minimap2
racon
medaka
trimgalore
bwa_align
pilon
quast
e
% single core CPU usage
0
500
1000
1500
2000
2500
kraken_db build
Guppy basecaller
pycoqc
fastp
filtlong
fastp trimmed
random subsetting
Kraken2
nanoplot
Raven
Miniasm
Canu
Flye
Krona
Raven_quast
Miniasm_quast
Canu_quast
Flye_quast
Trycycler
Minimap2
Racon
Medaka
Trimgalore
bwa_align
pilon
quast
f
Memory (Gb)
0
20
40
60
kraken_db build
Guppy basecaller
pycoqc
fastp
filtlong
fastp trimmed
random subsetting
Kraken2
nanoplot
Raven
Miniasm
Canu
Flye
Krona
Raven_quast
Miniasm_quast
Canu_quast
Flye_quast
Trycycler
Minimap2
Racon
Medaka
Trimgalore
bwa_align
pilon
quast
g
Time (mins)
0
200
400
600
800
1000
kraken_db build
Guppy basecaller
pycoqc
fastp
filtlong
fastp trimmed
random subsetting
Kraken2
nanoplot
Raven
Miniasm
Canu
Flye
Krona
Raven_quast
Miniasm_quast
Canu_quast
Flye_quast
Trycycler
Minimap2
Racon
Medaka
Trimgalore
bwa_align
pilon
quast
h
Read bytes (Gb)
0
100
200
300
400
kraken_db build
Guppy basecaller
pycoqc
fastp
filtlong
fastp trimmed
random subsetting
Kraken2
nanoplot
Raven
Miniasm
Canu
Flye
Krona
Raven_quast
Miniasm_quast
Canu_quast
Flye_quast
Trycycler
Minimap2
Racon
Medaka
Trimgalore
bwa_align
pilon

Figure S2. Summary information provided by Nextflow output for assembly of H37Rv using *in silico* data and H37Rv.1 comparing all 4 tested assemblers and polishing. a-d. H37Rv in silico data **a.** Amount of CPU usage **b.** Memory (RAM) usage **c.** Time to completion **d.** Input/Output (I/O) (how much data is read per task). **e-h.** H37Rv.1 data. **e.** Amount of CPU usage **f.** Memory (RAM) usage **g.** Time to completion **h.** Input/Output (I/O) (how much data is read per task). A single nextflow run of *in silico* (**a-d**) and *in vitro* data (**e-h**) respectively is shown.

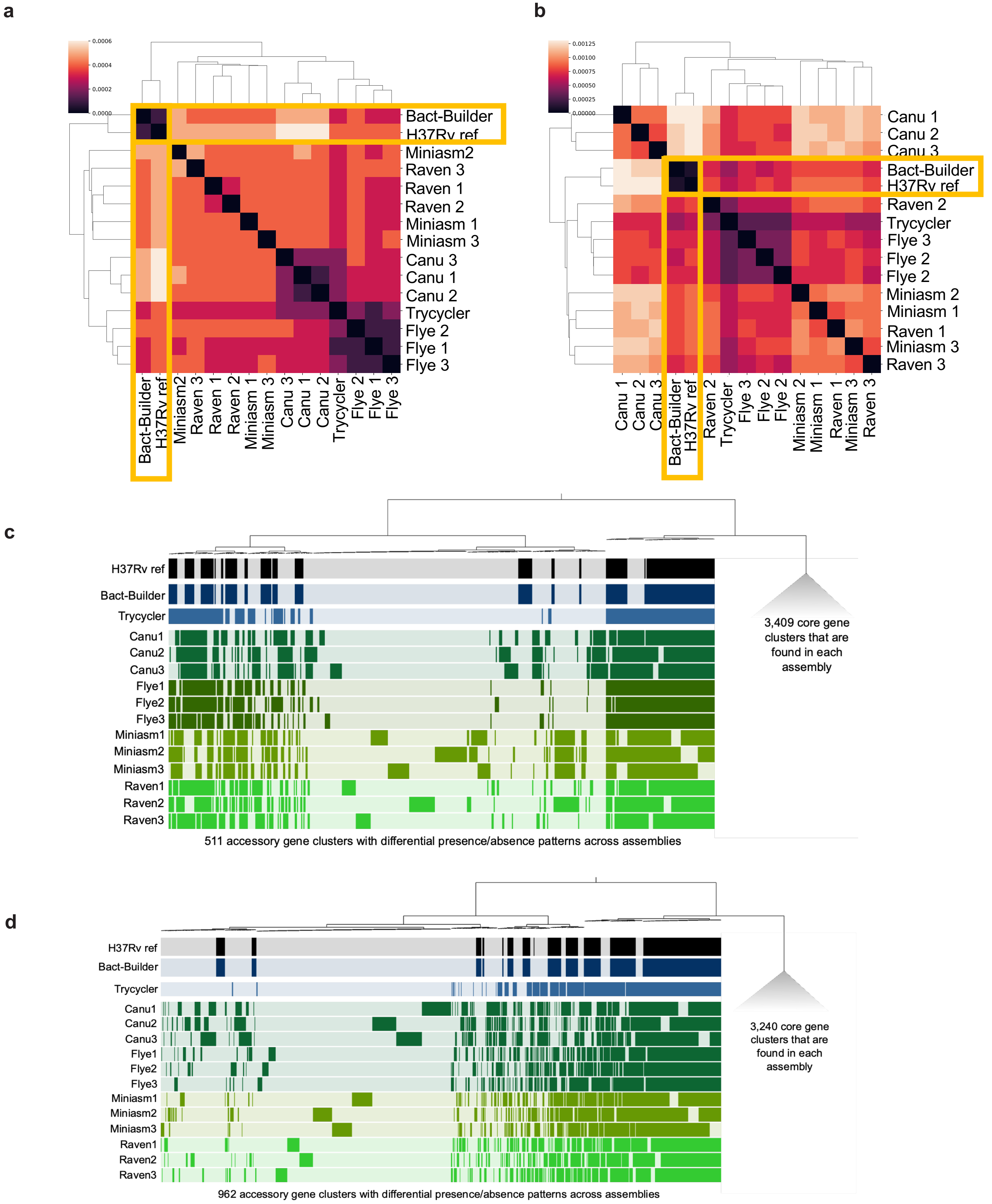

Figure S3. Comparing H37Rv.2 and H37Rv.3 assemblies. a-b. Heatmap of hierarchical clustering of the distance using euclidean average linkage clustering of differences between all assemblies for H37Rv.2 (**a**) and H37Rv.3 (**b**), the Bact-Builder output and the published reference (H37Rv ref) determined by DNAdiff. **c-d.** Anvi'o output showing differences in annotations between H37Rv.2 (**c**) and H37Rv.3 (**d**).

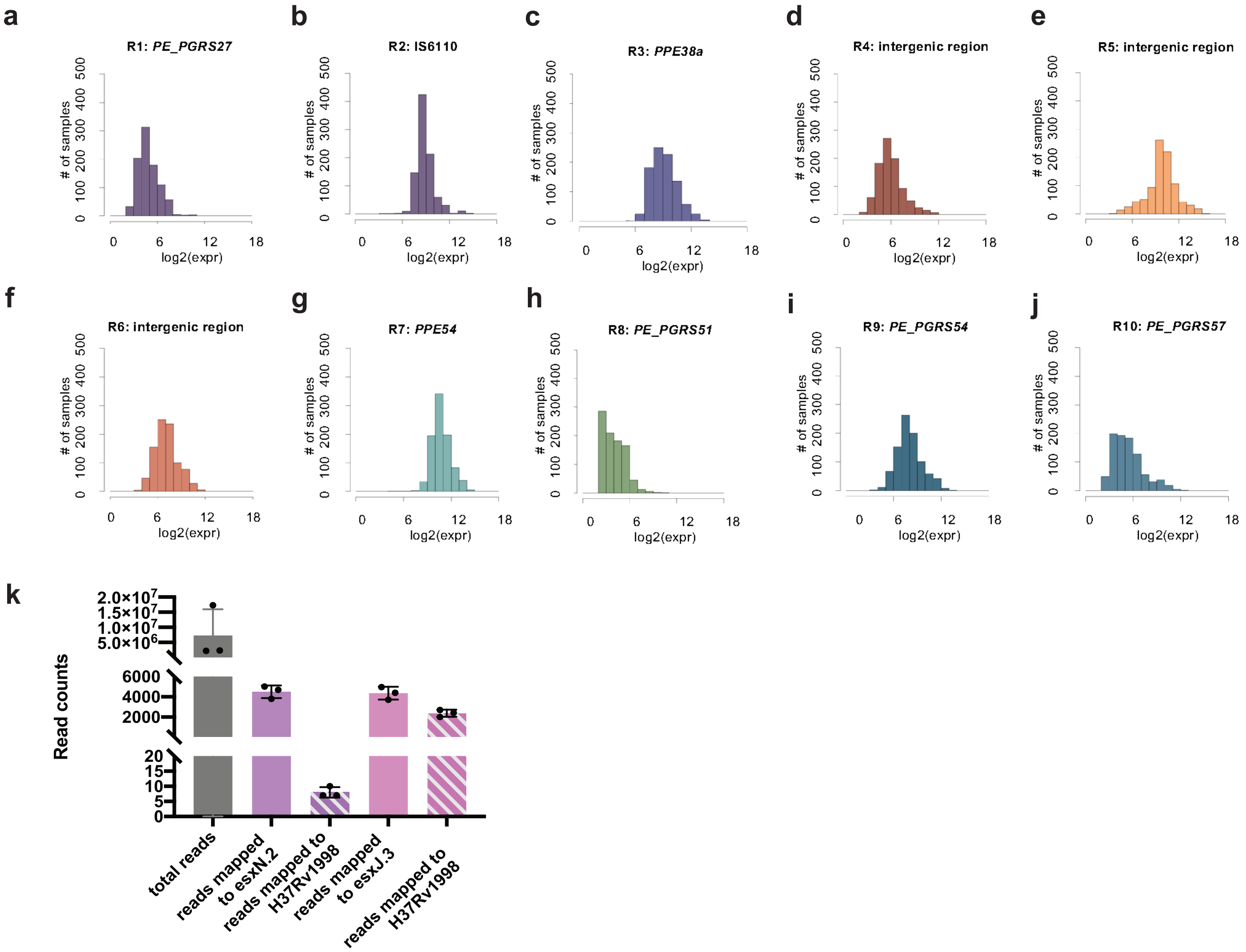

Figure S4. RNA counts of newly discovered genomic regions of difference from public RNA sequencing datasets. (**a-j**) Histogram of R1 – R10 respectively. Histograms demonstrate that all regions are expressed in H37Rv (new). **k**. Three independent RNAseq data sets were mapped to H37Rv(new) and H37Rv1998 using Bowtie2. Read coverage demonstrates that the majority of RNAseq reads that map to R3 do not map back to H37Rv1998. Read coverage is presented as mean $\pm$ SD.

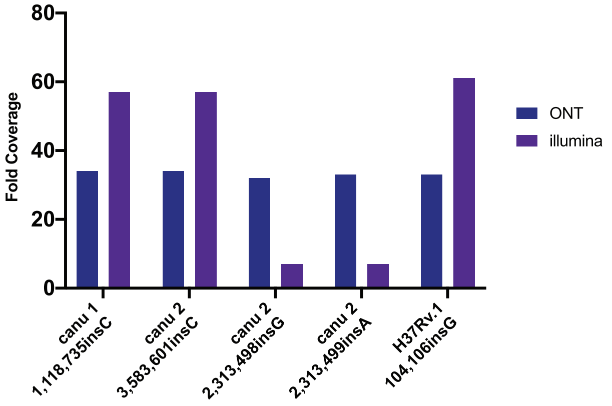

Figure S5. Evaluating ONT and illumina read coverage over identified indels. Minimap2 and Bowtie2 were used to align ONT and illumina data respectively to the polished Canu 1, Canu 2 and H37Rv.1 assemblies to evaluate the presence of indels indels.

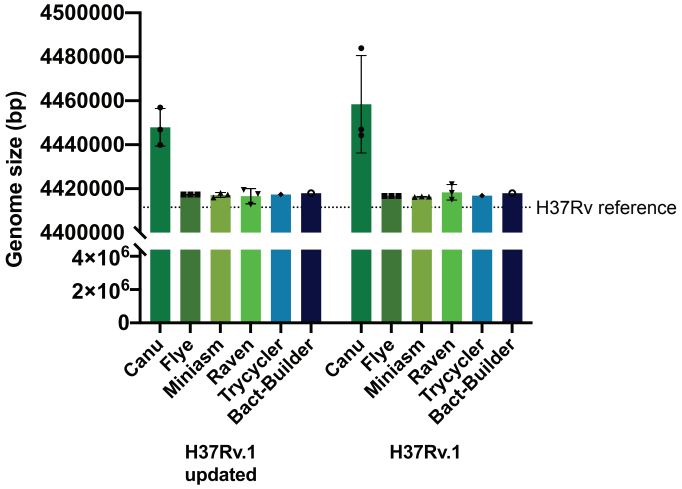

Figure S6. Evaluating updated Bact-Builder v1.1 versus Bact-Builder v1.0 programs. Relevant individual components of Bact-Builder v1.0 (Guppy, Canu and Flye) were updated to v1.1 using the most recent releases at the time of writing (08/22/22). Assemblies, Trycycler and Bact-Builder v1.1 outputs were compared against program versions used in Bact-Builder v1.0. Individual assemblers still demonstrate variability, however the final Bact-Builder v1.0 and 1.1 genomes were structurally identical and differed by a single nucleotide. All assemblies were run in triplicate as stated in the Bact-Builder methods and data is presented as mean ± SD

Supplementary References

1. Wick, R. Badread: simulation of error-prone long reads. *J Open Source Softw* **4**, 1316 (2019).

2. Huang, W., Li, L., Myers, J. R. & Marth, G. T. ART: A next-generation sequencing read simulator. *Bioinformatics* **28**, 593–594 (2012).

3. Hall, M. B. Rasusa: Randomly subsample sequencing reads to a specified coverage. *J Open Source Softw* **7**, 3941 (2022).

4. Schwengers, O. *et al.* ASA3P: An automatic and scalable pipeline for the assembly, annotation and higher-level analysis of closely related bacterial isolates. *PLoS Comput Biol* **16**, e1007134 (2020).

5. Petit, R. A. & Read, T. D. Bactopia: a Flexible Pipeline for Complete Analysis of Bacterial Genomes. (2020) doi:10.1128/mSystems.00190-20.

6. de Maio, N. *et al.* Comparison of long-read sequencing technologies in the hybrid assembly of complex bacterial genomes. *Microb Genom* **5**, (2019).

7. Goldstein, S., Beka, L., Graf, J. & Klassen, J. L. Evaluation of strategies for the assembly of diverse bacterial genomes using MinION long-read sequencing. *BMC Genomics 2019 20:1* **20**, 1–17 (2019).

8. Eren, A. M. *et al.* Community-led, integrated, reproducible multi-omics with anvi'o. *Nat Microbiol* **6**, 3–6 (2021).

9. Marçais, G. *et al.* MUMmer4: A fast and versatile genome alignment system. *PLoS Comput Biol* **14**, e1005944 (2018).

10. Wick, R. R. *et al.* Trycycler: consensus long-read assemblies for bacterial genomes. *bioRxiv* 2021.07.04.451066 (2021) doi:10.1101/2021.07.04.451066.

11. Walker, B. J. *et al.* Pilon: An integrated tool for comprehensive microbial variant detection and genome assembly improvement. *PLoS One* **9**, 112963 (2014).
