# Supplementary material for: A comprehensive update to the Mycobacterium tuberculosis H37Rv reference genome
Source: Nat Commun. 2022 Nov 18;13:7068. doi: 10.1038/s41467-022-34853-x (PMC9673877; doi:10.1038/s41467-022-34853-x)
Supplement: Supplementary file 3 — Description of Additional Supplementary Files [file 41467_2022_34853_MOESM3_ESM.pdf]

Description of Additional Supplementary Files:

Supplementary Data 1: SNP and indel differences found between the H37Rv1998 reference and H37Rv (new)

Supplementary Data 2: H37Rv(new) Fasta sequence

Supplementary Data 3: H37Rv(new) annotated file

Supplementary Data 4: Primer sequences for PCR validation of the R3 region
