# Supplementary material for: A comprehensive update to the Mycobacterium tuberculosis H37Rv reference genome
Source: Nat Commun. 2022 Nov 18;13:7068. doi: 10.1038/s41467-022-34853-x (PMC9673877; doi:10.1038/s41467-022-34853-x)
Supplement: Supplementary file 8 — Reporting Summary [file 41467_2022_34853_MOESM8_ESM.pdf]

nature portfolio

Corresponding author(s): David Alland

Last updated by author(s): 10/31/2022

Reporting Summary

Nature Portfolio wishes to improve the reproducibility of the work that we publish. This form provides structure for consistency and transparency in reporting. For further information on Nature Portfolio policies, see our Editorial Policies and the Editorial Policy Checklist.

Statistics

For all statistical analyses, confirm that the following items are present in the figure legend, table legend, main text, or Methods section.

n/a	Confirmed	
☐	☒	The exact sample size (*n*) for each experimental group/condition, given as a discrete number and unit of measurement
☐	☒	A statement on whether measurements were taken from distinct samples or whether the same sample was measured repeatedly
☐	☒	The statistical test(s) used AND whether they are one- or two-sided<br>*Only common tests should be described solely by name; describe more complex techniques in the Methods section.*
☒	☐	A description of all covariates tested
☒	☐	A description of any assumptions or corrections, such as tests of normality and adjustment for multiple comparisons
☐	☒	A full description of the statistical parameters including central tendency (e.g. means) or other basic estimates (e.g. regression coefficient) AND variation (e.g. standard deviation) or associated estimates of uncertainty (e.g. confidence intervals)
☐	☒	For null hypothesis testing, the test statistic (e.g. *F*, *t*, *r*) with confidence intervals, effect sizes, degrees of freedom and *P* value noted<br>*Give P values as exact values whenever suitable.*
☒	☐	For Bayesian analysis, information on the choice of priors and Markov chain Monte Carlo settings
☒	☐	For hierarchical and complex designs, identification of the appropriate level for tests and full reporting of outcomes
☒	☐	Estimates of effect sizes (e.g. Cohen's *d*, Pearson's *r*), indicating how they were calculated

Our web collection on statistics for biologists contains articles on many of the points above.

Software and code

Policy information about availability of computer code

Data collection	As described in the methods section, all genomic DNA samples were collected in-house and sequenced using the ONT MinION and illumina NovaSeq (paired end 2x150) platform.
Data analysis	As described in the methods section, all data analysis used in this paper used open source software. MinION sequencing was run using the MinKNOW v 5.0.5 program available through Oxford Nanopore Technologies (ONT). All packages used are listed in the methods section of the paper. Info on Bact-Builder and software versions can be found here: https://github.com/alemenze/bact-builder. Scripts used for RNAseq analysis can be found here: https://github.com/as2654/rna-seq-tb0/tree/main. DNAdiff and resulting figures were generated using CONCOCT: https://github.com/BinPro/CONCOCT. Pan-genomic analysis was run using: https://github.com/merenlab/anvio.

For manuscripts utilizing custom algorithms or software that are central to the research but not yet described in published literature, software must be made available to editors and reviewers. We strongly encourage code deposition in a community repository (e.g. GitHub). See the Nature Portfolio guidelines for submitting code & software for further information.

Data

Policy information about availability of data

All manuscripts must include a data availability statement. This statement should provide the following information, where applicable:

- Accession codes, unique identifiers, or web links for publicly available datasets
- A description of any restrictions on data availability
- For clinical datasets or third party data, please ensure that the statement adheres to our policy

Whole Genome Sequencing data for the strains used in this study is available from the NCBI Sequence Read Archive via BioProject accession number PRJNA83678. Updated annotation files have been deposited in GenBank, with primary accession code: SUB11453622 (GenBank approval pending). The fasta sequence and annotated file for H37Rv(new) can also be found in Supplementary files. RNAseq data was obtained via BioProject accession number (PRJNA507615) . The H37Rv1998 reference sequence was obtained from Genbank (NC_000962.3).

Human research participants

Policy information about studies involving human research participants and Sex and Gender in Research.

Reporting on sex and gender	NA
Population characteristics	NA
Recruitment	NA
Ethics oversight	NA

Note that full information on the approval of the study protocol must also be provided in the manuscript.

Field-specific reporting

Please select the one below that is the best fit for your research. If you are not sure, read the appropriate sections before making your selection.

☒ Life sciences ☐ Behavioural & social sciences ☐ Ecological, evolutionary & environmental sciences

For a reference copy of the document with all sections, see nature.com/documents/nr-reporting-summary-flat.pdf

Life sciences study design

All studies must disclose on these points even when the disclosure is negative.

Sample size	5 independent isolates of H37Rv were used in this study: Lab isolate, TMC102, TMC301, NR-123, and 1998H37Rv. Sample size was determined using all of the WT H37Rv samples available at ATCC in addition to our in-house stocks. This collection constitutes all WT H37Rv samples used globally
Data exclusions	No data was excluded from the analysis
Replication	All isolates of H37Rv were successfully extracted and sequenced in triplicate in this study
Randomization	This study involved direct comparisons between established H37Rv strains therefore randomization does not apply.
Blinding	This study involved direct comparisons between established H37Rv strains therefore blinding does not apply.

Reporting for specific materials, systems and methods

We require information from authors about some types of materials, experimental systems and methods used in many studies. Here, indicate whether each material, system or method listed is relevant to your study. If you are not sure if a list item applies to your research, read the appropriate section before selecting a response.

Materials & experimental systems

n/a	Involved in the study
☒	☐ Antibodies
☒	☐ Eukaryotic cell lines
☒	☐ Palaeontology and archaeology
☒	☐ Animals and other organisms
☒	☐ Clinical data
☒	☐ Dual use research of concern

Methods

n/a	Involved in the study
☒	☐ ChIP-seq
☒	☐ Flow cytometry
☒	☐ MRI-based neuroimaging
